# The use of tantalum cones and diaphyseal-engaging stems in tibial component revision: a consecutive series

**DOI:** 10.1186/s43019-022-00141-7

**Published:** 2022-03-10

**Authors:** P. Spinello, R. A. Ruberte Thiele, K. Zepeda, N. Giori, P. F. Indelli

**Affiliations:** 1 Department of Orthopaedic Surgery, Stanford University School of Medicine, PAVAHCS – Surgical Services, 1801 Miranda Ave, Palo Alto, CA 94304 USA; 2 Touro College of Osteopathic Medicine, New York, NY USA; 3 Department of Orthopaedic Surgery, University of Rome “La Sapienza”, Rome, Italy

## Abstract

**Introduction:**

Revision knee arthroplasty presents a number of challenges, including management of bone loss. The goal in managing moderate to large bone defects is fixation that is sufficient enough to allow early weight-bearing. The purpose of this study was to describe the surgical technique and clinical and radiographic outcomes of patients treated with porous tantalum metaphyseal cones in combination with long uncemented diaphyseal-engaging stems to manage tibial bone loss in revision total knee arthroplasty (TKA).

**Materials and methods:**

Thirty-six aseptic revision TKAs were performed at our institution between 2016 and 2019 by two senior authors. A single trabecular metal tantalum cone combined with a long (100 or 155 mm) press fit, diaphyseal-engaging stem was used in all cases to reconstruct metaphyseal bone defects and to augment tibial fixation. Cemented stems were excluded. The tibiofemoral angle was measured along the tibial and femoral shaft axes on the weight-bearing anteroposterior radiograph at final follow-up (range 15–56 months). All clinical and surgical complications, reoperations, and revisions of any component were recorded. Survivorship free of revision was evaluated at the time of the latest follow-up.

**Results:**

The mean Knee Society Score (KSS) and Knee Society Function Score (KSS-F) improved significantly from 29.7 points preoperatively (range 11–54 points) to 86 points (range 43–99 points) and from 20.4 points preoperatively (range 0–55 points) to 72.3 points (range 30–90 points) (*p* < 0.05), respectively. Eleven tibial constructs (30.5%) had incomplete, nonprogressive radiolucent lines (≤ 2 mm). All tibial cones demonstrated osteointegration. One patient underwent a full revision for periprosthetic joint infection, and survivorship free of any component revision was 91.7% at final follow-up.

**Conclusions:**

Hybrid fixation with uncemented diaphyseal-engaging stems and porous tantalum metaphyseal cones resulted in radiographic lack of osteolysis, good clinical outcomes, and survivorship of 91.7% at a median follow-up of 33 months when considering all-cause revision as the endpoint.

## Background

While instability, infection, and stiffness represent the main causes of TKA failure [1], other reasons for TKA failure include aseptic loosening, osteolysis, periprosthetic fracture, extensor mechanism complications, and chronic pain [2]. Revision knee arthroplasty presents a number of challenges, including bone loss and ligamentous deficiency. The ability to achieve longitudinal alignment [3], adequate fixation [4], and postoperative stability has been related to increased survivorship [5]. Addressing moderate to large bone defects should result in solid fixation to allow early weight-bearing. Current options to achieve such initial fixation include cemented components, impaction bone grafting, bulk allografts, traditional metal augments, and, more recently, metaphyseal sleeves and porous tantalum metaphyseal cones. Bone defects are historically divided into three types according to the Anderson Orthopedic Research Institute (AORI) classification [6]: Type 1 defect (intact metaphyseal bone) refers to minor bone defects that will not compromise the stability of a revision component and can generally be managed with cementing techniques, bone grafting, and with or without screws. Type 2 defect (damaged metaphyseal bone) refers to loss of cancellous bone in the metaphyseal segment and it is further subdivided, with type 2A defects affecting only one femoral or tibial condyle and type 2B defects involving both femoral or tibial condyles. In general, type 2A defects can be managed with addition of metal augments or bone graft, and type 2B with structural grafts and/or metal filling devices like sleeves and cones. Last, type 3 defect (deficient metaphyseal segment) refers to bone loss that comprises a major portion of either condyle or plateau, and it is occasionally associated with collateral or patellar ligament detachment. Type 3 defects have been historically treated with structural grafts and/or sleeves or cones. The addition of stems (whether cemented or uncemented) is generally thought to minimize the strain at the bone–implant interface.

Once the defect has been quantified, solid fixation should be obtained. Morgan-Jones et al. [7] introduced the “zonal fixation theory.” The distal femur and proximal tibia were divided into three anatomical zones: zone 1, the joint surface or epiphysis; zone 2, the metaphysis; and zone 3, the diaphysis. The authors [7] suggested that, in a TKA revision scenario, solid fixation should be obtained in at least two of the three zones. The current authors have historically managed large, tibial bone defects by applying the principles of a hybrid fixation, using diaphyseal-engaging stems combined with metaphyseal, tantalum cones: there are multiple potential advantages of this technique, including better tibial component alignment, improved osteointegration of the tantalum cones with respect to structural allografts, and achievement of a final, rigid construct that avoids postoperative stem migration.

The purpose of this study was to describe the surgical technique and to determine the clinical and radiographic outcomes of patients treated with porous tantalum metaphyseal cones in combination with long uncemented diaphyseal-engaging stems to manage tibial bone loss in revision TKA. At a median follow-up of 33 months, this single-institution experience focuses on clinical scores, radiographic evidence of osteointegration, and complications.

## Materials and methods

This is a single-center retrospective study of a consecutive series of aseptic revision TKA performed at our institution between 2016 and 2019 by two senior authors (NG, PI). Indications for revision included instability with associated bone loss, second-stage reimplantation for periprosthetic joint infection (PJI), loosening of the tibial component, severe tibial osteolysis in the presence of a well-fixed tibial component, and stiffness (Table 1). A single trabecular metal tantalum cone combined with a long (100 or 155 mm) press fit, diaphyseal-engaging stem (Zimmer Biomet, Warsaw, IN, USA) was used in all cases to reconstruct metaphyseal bone defects (AORI 2 or greater) and to improve tibial fixation. Patients with cemented stems were excluded.

**Table 1:** Demographical data, indication for surgery, and classification of the tibial bone defect according to the Anderson Orthopedic Research Institute (AORI) classification

Age	BMI	Reason for revision	AORI classification
65.89	31.646	39% (14/36) aseptic loosening 39% (14/36) PJI 17% (6/36) instability 5% (2/36) stiffness	50% (18/36) type 2B 28% (10/36) type 2A 22% (8/36) type C

A total of 36 patients (35 males, 1 female; mean age 65.8 years at time of surgery) were included. Characteristics of the cohort, including AORI classification, demographics, body max index (BMI), and indications for surgery, were recorded (Table 1): 36% of patients (13 patients) had an immediate or prior history of PJI. All patients had inflammatory markers [erythrocyte sedimentation rate (ESR) and C-reactive protein (CRP)] measured preoperatively to rule out occult infection. All patients had a minimum clinical and radiological follow-up of 1 year (range 15–56 months).

### Clinical outcome

Knee function was assessed preoperatively, postoperatively, and at final follow-up with the use of the Knee Society Score (KSS) and Knee Society Function Score (KSS-F) [8, 9]. All clinical and surgical complications, reoperations, and revisions of any component were recorded.

### Radiological evaluation

Anteroposterior and lateral films from the immediate postoperative period were reviewed and compared with the latest follow-up to assess the integrity of the tibial stem–cone construct [10]. The canal fill ratio (CFR: width of the stem divided by the width of the intramedullary canal), which has been described as a predictor of proper mechanical alignment and implant survival [3], was measured at 1.5 cm proximal to the stem tip on the postoperative (6 weeks) weight-bearing anteroposterior radiograph for all stems [11]. The ideal CFR to achieve a stable intramedullary fit [3] has been defined as > 0.85. The tibiofemoral angle was measured along the tibial and femoral shaft axes on the weight-bearing anteroposterior radiograph at final follow-up. Since osteolysis has an insidious onset, it is often asymptomatic, and lesions are often detected incidentally on follow-up radiographs, the authors always obtained, when an osteolysis was suspected, at least two orthogonal views to visualize the area.

### Surgical technique

The surgical technique used in this study was similar to previous reports of revision knee arthroplasty [11]: the current authors, after implant removal, assessed the extension of the tibial bone defect after debridement of nonviable bone and osteolytic lesions if present.

**Fig. 1:**
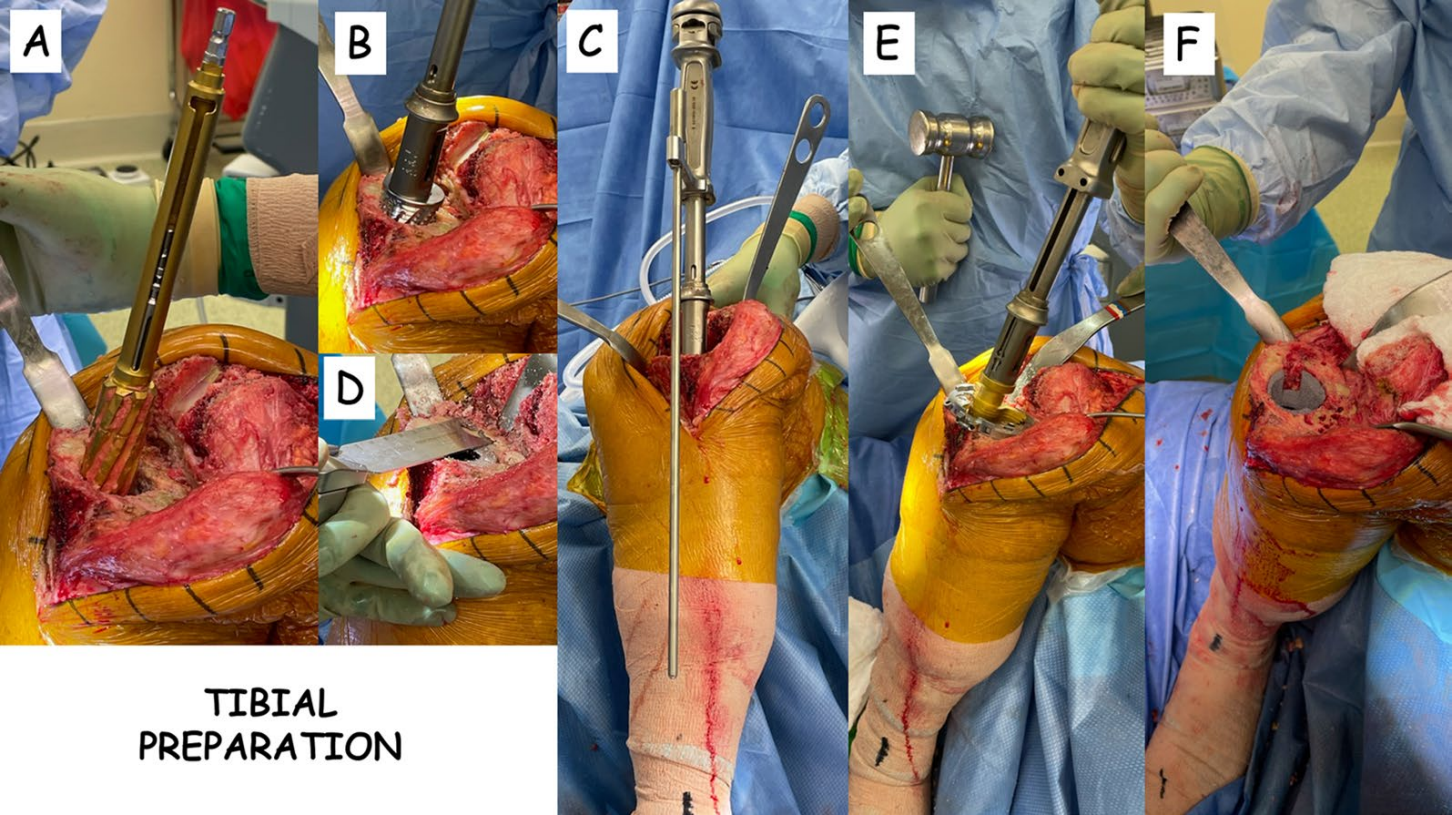
**A** Preparation for the tibial cone: bone reaming; **B** preparation for the tibial cone: cone broach; **C** extramidollary alignment check; **D** tibial recut using the cone broach as a reference for correct varus/valgus and slope alignment; **E** final tibial preparation; **F** placement of the tibial cone

The AORI classification [6] was used to identify patients who required a porous tantalum metaphyseal cone and the Morgan-Jones classification [7] was used to recognize the two anatomical zones in which fixation needed to be achieved. The type of bone loss registered in this consecutive series is presented in Table 1. Once the quality of bone loss was determined, a series of flexible reamers were introduced in line with the tibial medullary canal. Once the tibial canal preparation using flexible reamers was completed, a series of implant-specific straight reamers were used to establish the adequate stem/bone engagement for the diaphyseal stem [12]. The final straight reamer always had solid engagement in the tibial diaphysis. The reamer handle was then removed, and a custom cone-preparing reamer was used to initiate metaphyseal preparation for the cone (Fig. 1A). At this point, cone size-specific broaches were used for the final impaction of the porous tantalum cones, still using the diaphyseal-engaging straight reamer as an intramedullary alignment guide to place the broach (Fig. 1 B). The overall alignment of the tibial construct was then checked (Fig. 1C). At this point, the cone impactor handle was removed, and the cone broach was used as a reference for the tibial cut to obtain the desired varus/valgus and slope alignment (Fig. 1D). The straight reamer was then removed, and the trial components (stem and tibial baseplate) were placed in the tibia to determine the stability and the alignment of the construct: if necessary, an offset stem was used to achieve better anteroposterior or mediolateral coverage of the tibial plateau. In this consecutive series, tibial offset stems were used in 19.4% of the knees. Once the stability and the alignment were found to be satisfactory, the final preparation of the tibia was performed (Fig. 1E). After pulsatile irrigation with normal saline, the final tibial cone was impacted into bone (Fig. 1F); the internal surface of the cone provided a receptive surface for the cementation of the tibial implant. The authors followed a hybrid technique [13] where the articular and metaphyseal portions of the final implant were cemented on the joint surface and inside the tantalum cone hand-packing the tibial keel with cement, and the diaphyseal-engaging portion of the stem was uncemented. Antibiotic-added cement (Palacos R + G, Heraeus, Hanau, Germany) was used in all knees: again, the cement was placed between the porous cone and the tibial tray and the proximal keel of the tibial component to unitize the stemmed tibial implant and the porous cone. The authors used only 100 and 155 mm slotted, titanium diaphyseal-engaging stems (Zimmer Biomet, Warsaw, IN, USA) in this series [14]: offset stems were used in 19.4% of knees.

### Statistical analysis

Demographic characteristics were analyzed descriptively. Continuous variables were compared between pre-intervention and post-intervention values with the use of a paired *t*-test. Statistical significance was set at *p* < 0.05. The survival of the implants was defined as the percentage of components (total and tibial only) that were still in place at the time of the latest follow-up.

## Results

A total of 36 patients (35 males, 1 female) were ultimately enrolled in the study at a mean follow-up of 33 months (range 17–58 months): one patient underwent explant because of a periprosthetic joint infection that occurred after 15 months from the revision surgery. There were 14 patients (38%) with a BMI > 35 kg/m^2^. All 14 patients (38%) who underwent revision surgery following a PJI did not have their patella resurfaced at the time of follow-up. The surgeons used a varus–valgus constrained (VVC) implant in 34 knees (94%), a posterior-stabilized (PS) implant in 1 knee, and a hinged implant in another knee.

The reasons for revision surgery were aseptic loosening (14 cases, 38%), second-stage reimplantation following a PJI (13 cases, 36%), one-stage reimplantation following acute PJI (1 case, 2.7%), instability (6 cases, 16%), and stiffness (2 cases, 5.5%). Bone loss was classified according to the AORI classification [6] as presented in Table 1. On the tibial side, augments were used in 19% of the cases and tantalum cones were used in 100% (small 31%, medium 50%, large 19%). A press-fit tibial stem was used in all cases: the most commonly used stem was the 155-mm-long one (83%); in 17%, a 100-mm-long stem was used. In 19.4% of the cases, an offset stem was used.

### Clinical results

The preoperative range of motion (ROM) consisted of a mean flexion contracture of 4° (range 0–20°) and a mean flexion of 80° (range 15–120°). At the time of the latest follow-up, knee motion had improved to a mean residual flexion contracture of 0.6° (range 0–5°) and to a mean flexion of 111.9° (range 90–130°) (*p* < 0.05). The mean clinical Knee Society Score improved significantly from 29.7 points preoperatively (range 11–54 points) to 86 points (range 43–99 points). The mean Knee Society Function Score improved significantly from 20.4 points preoperatively (range 0–55 points) to 72.3 points (range 30–90 points) (*p* < 0.05).

### Radiographic results

**Fig. 2:**
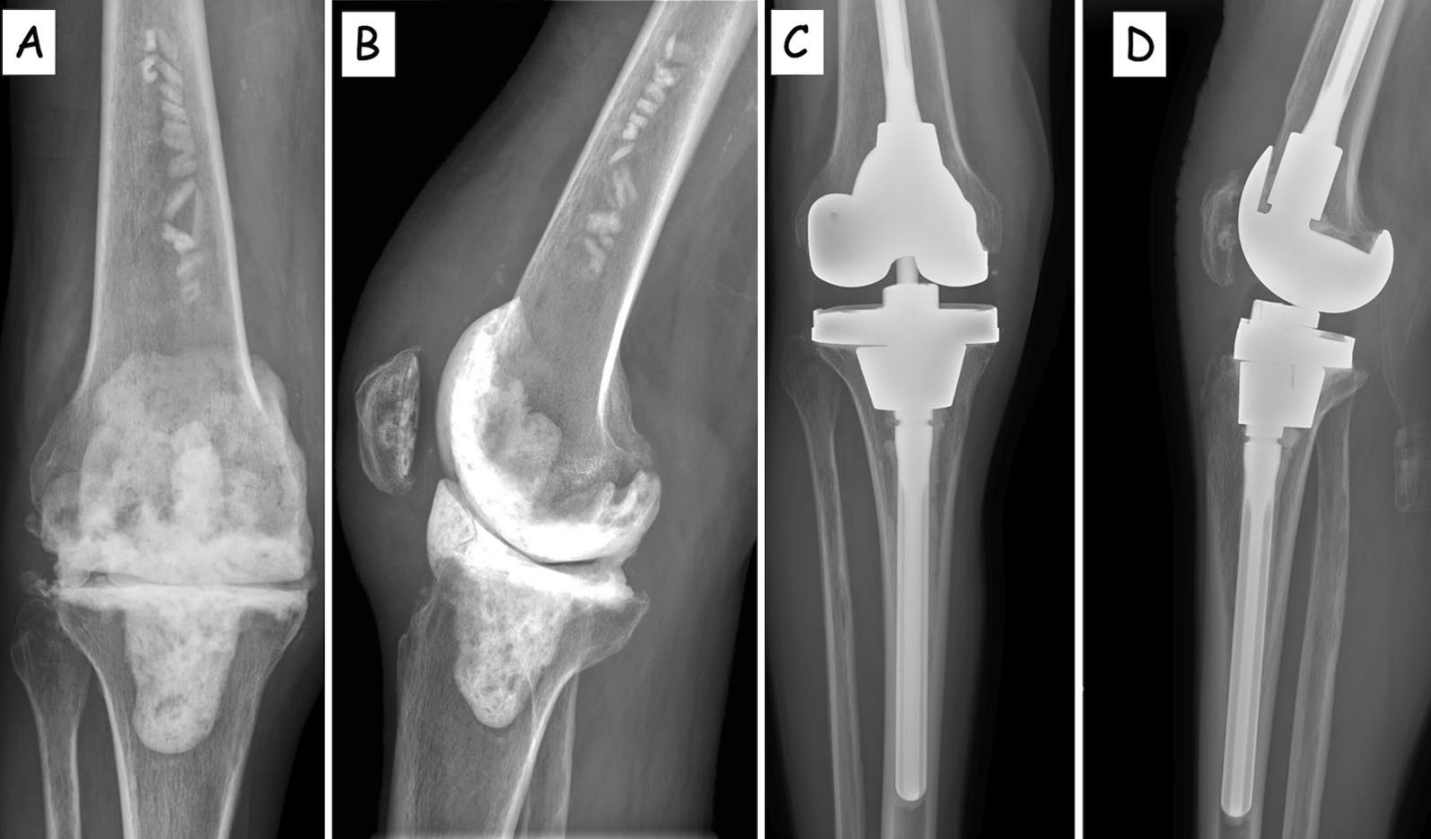
Right knee: 69-year-old patient. **A** Anteroposterior view of the knee: dynamic spacer in place following a periprosthetic joint infection (PJI); **B** lateral view of the knee: dynamic spacer in place following a periprosthetic joint infection (PJI); **C** and **D** anteroposterior and lateral views of the knee at 2 years follow-up: the tibial cone is well integrated

**Fig. 3:**
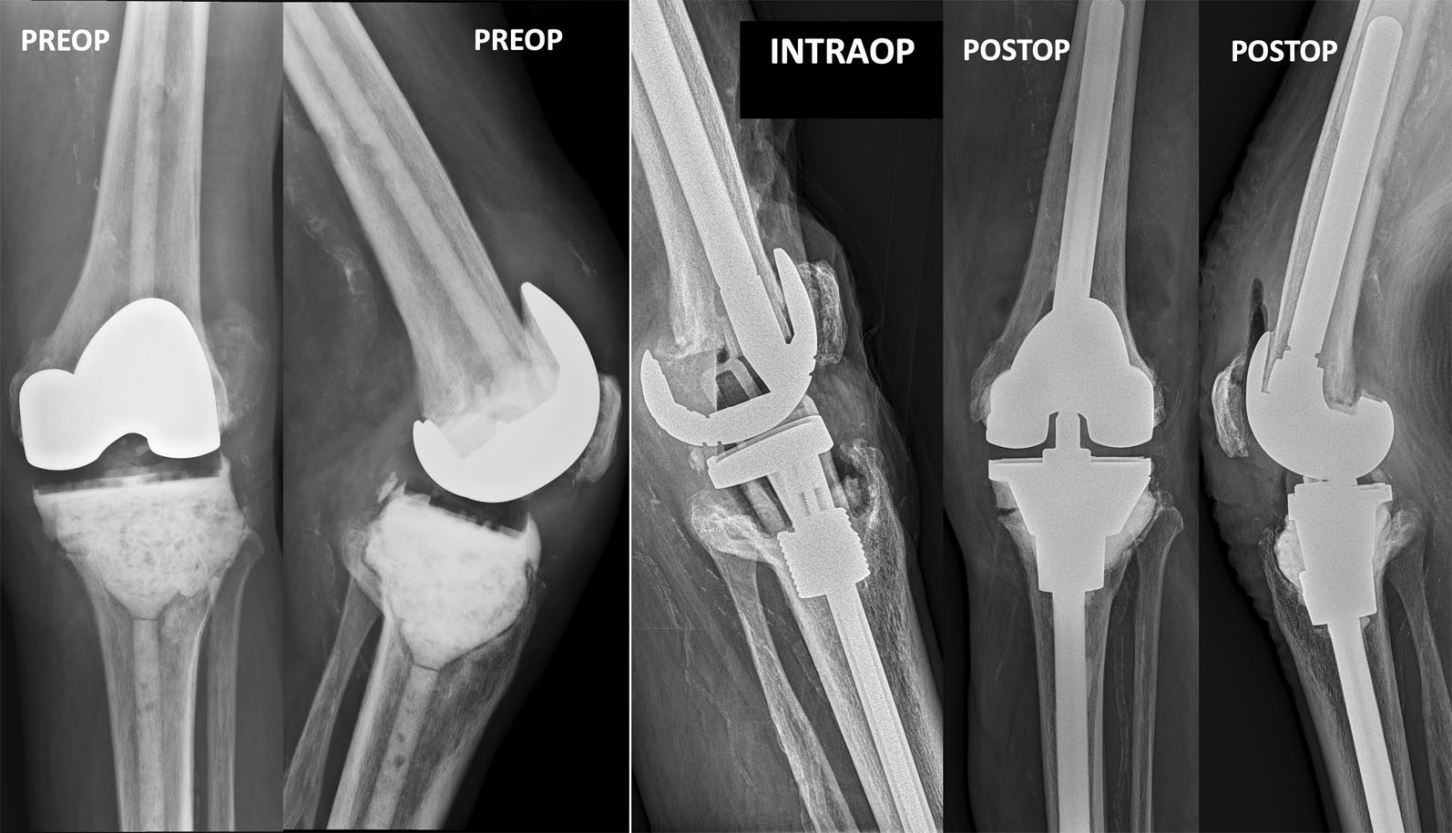
Seventy-eight-year-old patient with a right knee dynamic spacer following a periprosthetic joint infection (PJI). Left: preoperative anteroposterior and lateral radiographs. Center: intraoperative lateral radiograph showing alignment of the trial components and amount of bone loss (Anderson Orthopaedic Research Institute Knee Bone Loss Classification—AORI 3) [6]. Right: postoperative anteroposterior and lateral radiographs, showing stacked trabecular metal cones (small and large) on the tibia

The mean preoperative tibiofemoral alignment was 7.6° varus (range 20° varus to 17° valgus), which improved to 6.4° valgus (range 3° varus to 9° valgus). Eleven tibial constructs (30.5%) had incomplete, nonprogressive radiolucent lines (≤ 2 mm) at the tibial baseplate bone–cement interface, mostly located on zone 3–4 on the AP view (11/11) and on zone 1–2 on the AP view (2/11). All tibial cones demonstrated osteointegration, as evidenced by reactive trabeculae formation at the points of cone–host bone contact (Figs. 2, 3): none of the cones was subsided at the time of follow-up. The tibial canal fill ratio (CFR) was measured in all cases: mean CFR on AP radiographs was 88% (range 70–96%).

### Complications

Six patients (16.6%) had complications during the study period: four of them (11.1%) had complications related to the surgical technique. Two patients (5.5%) had a periprosthetic joint infection (PJI). One patient had a subacute PJI at 10 weeks from the original surgery: the microorganism was isolated, and the patient underwent Debridement, Antibiotic Pearls, Retention of the Implant (DAPRI) procedure [15] and a course of 12 weeks of antibiotic therapy; he was asymptomatic, and he had normal PJI serologic markers at the time of the latest follow-up (23 months). One patient underwent revision involving explantation at 15 months from the original surgery because of chronic PJI: his original surgery was a two-stage revision following a previous PJI. Two patients had aseptic, mechanical complications: one patient underwent a polyethylene liner exchange at 36 months from the original revision surgery because of early loosening of the polyethylene insert due to failure of its locking mechanism; one patient was found to have a radiographically loose femoral component with moderate clinical symptoms, and he is scheduled for revision of the femoral component, which has been delayed due to coronavirus disease 2019 (COVID-19) restrictions: this was considered as aseptic loosening of the femoral component. Two patients (5.5%) had intraoperative complications. One patient had an intraoperative partial avulsion of the patellar tendon distal insertion: it was repaired using a #2 FiberWire (Arthrex, Naples, FL, USA) in a double tunnel technique; the patient used a postoperative brace for 6 weeks postoperatively and had a range of motion from 0° to 110° at final follow-up; one patient had an intraoperative fracture involving the posteromedial corner of the tibial plateau that required open reduction internal fixation (ORIF) with two 25 mm cancellous bone screws that were oriented obliquely to avoid contact with the stem and the cone. This patient followed a standard postoperative rehabilitation protocol with weight-bearing as tolerated from postoperative day 1.

### Survivorship

Survivorship free of any component revision was 91.7% at the time of the latest follow-up (mean 31 months, range 15–56 months). Survivorship free of revision of the tibial cone/tibial stem construct was 97.3% at the time of the latest follow-up.

## Discussion

This study showed that hybrid fixation with uncemented diaphyseal-engaging stems and porous tantalum metaphyseal cones has good clinical outcomes and survivorship of 92% at a mean follow-up of 31 months. Our results are similar to those reported in previous studies on a hybrid technique that reported an overall survival of 90% (range 83–98%) at a similar follow-up. The main characteristics of the previous studies on hybrid fixation are summarized in Table 2: the rate of complications of the current study did not differentiate significantly from the current literature. The number of patients undergoing revision TKA continues to grow [16]. In our experience, the use of long diaphyseal-engaging stems, combined with the tantalum metaphyseal cones in tibial component revision surgery, provides a stable construct and a satisfactory clinical outcome.

**Table 2:** Comparison between the current study and previous studies

Study	Knees	Follow-up (months)	Rate of survival rTKA	Preoperative clinical score	Postoperative clinical score	ROM preoperative	ROM postoperative	Radiographic findings on tibial component	Complications
*Haas *et al. *1995*	67	42	83%	49 (KSS)	76 (KSS)	80°	95°	**4%** radiolucent lines. Complete but nonprogressive **69%** radio-opaque lines along tibial rods	**13%** (10/67) Intraoperative fracture, rupture of quadriceps tendon, wound complication, postoperative dislocation, patellectomy, hardware removal, infection, stiffness
*Gofton *et al. *2002*	89	71	94%	85 (KSS)	133 (KSS)	88°	98°	Common incomplete radiolucent lines **6%** (5/89) complete radiolucent lines that led to 5 revisions **52%** radio sclerotic lines near press-fit stem	**8%** (7/89) postoperative hematoma, quadriceps turndown, patellar clunk syndromes, femoral stem disengagement, tibial tubercle osteotomy migration
*Shannon *et al. *2003*	63	69	81%	56 (KSS) 49 (KSS-F)	81 (KSS) 62 (KSS-F)	n/a	n/a	**57%** radiolucent lines **6%** (4/63) cases with tibial loosening signs **97%** incomplete parallel sclerotic lines around the stem	**16%** (10/63) aseptic loosening, heterotopic ossification, patellar osteolysis, wound complication
*Bottner *et al. *2006*	33	38	94%	42 (KSS) 48 (KSS-F)	83 (KSS) 76 (KSS-F)	94°	105°	**3%** (1/33) complete progressive radiolucent lines	**6%** (2/33) aseptic loosening
*Wood *et al. *2008*	135	24	98%	38 (KSS) 32 (KSS-F)	86 (KSS) 55 (KSS-F)	87°	108°	**11%** not progressive tibial radiolucent lines **2%** (3/13) progressive radiolucent lines **90%** partial or complete radio sclerotic lines around stems, not progressive over time	**5%** (7/135) aseptic loosening, medial collateral ligament rupture, infection
*Peters *et al. *2009*	184	49	93%	135 (KSS) 63 (KSS-F)	168 (KSS) 82 (KSS-F)	n/a	n/a	**3%** incomplete radiolucent lines No complete radiolucent lines	**16%** (29/178) infections, stiffness polyethylene exchange, patellofemoral clunk syndrome
*Sah *et al. *2011*	88	65	84%	46 (KSS) 48 (KSS-F)	85 (KSS) 68 (KSS-F)	n/a	n/a	**19%** (17/88) partial and nonprogressive tibial radiolucent lines **1%** (1/88) radiographic loosening tibial component **75%** (66/88) radio sclerotic lines around tibial stems, predominantly at the tip	**10% (**9/88): aseptic loosening, infections, periprosthetic fracture
*Current study*	36	31	92%	30 (KSS) 20 (KSS-F)	86 (KSS) 72 (KSS-F)	76°	111*	**31%** (11/36) incomplete, nonprogressive radiolucent lines **3%** (1/36) radiographic loosening of tibial component	**17%** (6/36) infections, early polyethylene insert loosening, aseptic loosening

*KSS* Knee Society Score, *KSS-F* Knee Society Score Functional

Hybrid fixation is an established surgical technique, with more than 30 years of history [17]; the idea that long stems improve component stability has comprehensively been demonstrated in the literature [18, 19]. A salient improvement in clinical and functional scores was observed using this technique (Table 2); the sample analyzed in our study aligns with and confirms these findings. The reliability of hybrid fixation was assessed by comparing its outcomes with cemented fixation in experimental [20, 21] and clinical settings [22]. As shown by the recent meta-analysis of Wang et al., which analyzes the available studies on the comparison of these two techniques, no significant differences in failure for any reason, reoperation, aseptic loosening, or infection between the two techniques were observed. Sheridan et al. demonstrated that the use of hybrid stems in TKA revisions produced a better outcome than cemented stems [23]. On the other hand, the use of cement has its disadvantages in cases of re-revision. Among major concerns, bone stock depletion due to extraction of the prosthesis [24] and the risk of tibial component malalignment during the surgery [3, 24, 25] should be considered when this technique is used. Regarding hybrid technique, some key principles should be kept in mind, as Gililland et al. [26] suggested in their recent study. When using this technique, a press-fit stem should achieve a minimum of 4 cm of diaphyseal fit [19]. Regarding stem stability, the diameter of the stem should be considered in relation to the intramedullary canal; canal fill ratio (the stem diameter divided by the diameter of the intramedullary canal) should be > 0.85 to obtain a stable construct. The surgical technique used in the current study population clearly follows these rules: a 150 mm and 100 mm press-fit stem was used in 83% and 17% of cases, respectively, obtaining a mean CFR of 0.88. This study suggests that a combination of stem length, stem diameter, and intramedullary canal geometry may be key for the survivorship of the implant. Similarly, Fleischman et al. [27] recommended to maximize diaphyseal engagement with hybrid fixation by using long canal-filling press-fit stems and reaming appropriately to reach optimal interference fit.

Among other advantages, diaphyseal-engaging stems are critical to the management of bone loss [28] and provide for better component alignment during revision surgery [19]. In our sample, a mean improvement of tibiofemoral alignment was obtained, from 7.6° varus to 6.4° valgus, which represents an optimal target value for improving implant survival [29]. Furthermore, commercially available implants offer great modularity through offsets stem extensions that can be helpful in situations in which anatomical mismatch, malalignment, and gap balancing issues are encountered [30]. These alluring qualities have guided our choice to diaphyseal-engaging stems, regardless of the initial bone defect. The use of metaphyseal-engaging stems, especially when associated with cementless fixation, should be avoided in revision knee arthroplasty, however, as a worrisome rate of aseptic loosening and radiographic instability of the implants has been observed in the literature [31]. In our study, trabecular metal cones have been used to address metaphyseal bone defects instead of using structural allografts, making the most of the qualities of the former and avoiding the disadvantages of the latter. Among their advantages, tantalum cones are easier to implant compared with structural allograft and showed good osteointegration as demonstrated by osteoblast expression and osseous ingrowth [32, 33]. Jensen et al. [34] in their randomized radio-stereometric analysis affirmed that tantalum cones combined with diaphyseal-engaging stems on the tibia provide a rigid construct that avoids tibial stem migration, allowing perfect conditions for bone ingrowth and fixation of the prosthesis. In our sample, all tibial cones demonstrated osteointegration, as evidenced by reactive trabeculae formation at the points of cone–host bone contact. Regarding functional outcomes, few studies showed, in line with our findings, satisfactory early-to-midterm results with significant improvement when tantalum cones were used [35–38]. Furthermore, by using this relatively new technique, the risks associated with the use of structural allograft, such as graft resorption, disease transmission, nonunion, malunion, and collapse, can be avoided [39].

Lastly, our findings agree with the ones previously reported in the literature [Table 2] regarding the rate of radiolucent lines, although the average follow-up was different between the present and other studies analyzed: radiolucent lines, when present, tend to be incomplete, with nonprogressive trends, and do not seem to be related to any pathologic features or development of aseptic loosening. Only one patient was found to have a radiographically loose femoral component; no signs of aseptic loosening concerning the tibial implant were present at the latest follow-up.

This study has multiple limitations, including its retrospective design and the lack of a control group sample. The cases analyzed come from the two senior authors’ personal database and were not assessed by a blinded independent examiner. The relatively small sample size may have led to the extent of the variability being underestimated; however, the similarity between our outcomes and those reported in the literature suggests that the sample is sufficiently representative. A mid- to long-term follow-up is needed to determine whether the satisfactory clinical and radiographic short-term results persist over time.

## Conclusions

Hybrid fixation with uncemented diaphyseal-engaging stems and porous tantalum metaphyseal cones has shown good clinical outcomes and survivorship of 92% at a mean follow-up of 31 months. A salient improvement in clinical and functional scores was observed using this technique. Tantalum cones have been used to address metaphyseal bone defects and demonstrated radiographic signs of osteointegration, guaranteeing perfect conditions for bone ingrowth and fixation of the tibial implant.

## Data Availability

The data that support the findings of this study are available on request from the corresponding author.

## Abbreviations

TKA: Total knee arthroplasty
KSS: Knee Society Score
KSS-F: Knee Society Score Functional
AORI: Anderson Orthopedic Research Institute
CFR: Canal filling ratio
ROM: Range of motion
FU: Follow-up
